# Feeding preferences and nutritional niche of wild water buffalo (*Bubalus arnee*) in Koshi Tappu Wildlife Reserve, Nepal

**DOI:** 10.1002/ece3.6183

**Published:** 2020-06-23

**Authors:** Tej Kumar Shrestha, Lee J. Hecker, Achyut Aryal, Sean C. P. Coogan

**Affiliations:** ^1^ Lumbini Environmental Services Pvt. Ltd. Kathmandu Nepal; ^2^ Department of Renewable Resources University of Alberta Edmonton AB Canada; ^3^ CC Group Limited/CC Training Academy Auckland New Zealand; ^4^ Save Dot International Limited Auckland New Zealand

**Keywords:** *Bubalus arnee*, multidimensional nutritional niche, nutritional ecology, wild water buffalo

## Abstract

The nutritional characteristics of food resources play an important role in the foraging behavior of animals and can provide information valuable to their conservation and management. We examined the nutritional ecology of wild water buffalo (*Bubalus arnee*; hereafter “buffalo”) in the Koshi Tappu Wildlife Reserve of Nepal during autumn using a multidimensional nutritional niche framework. We identified 54 plant species as being foraged by buffalo. We found that buffalo consumed graminoids and forbs 2–3 times more frequently than browse items. Proximate analyses of the 16 most frequently foraged plants indicated that buffalo diets were highest in carbohydrate (40.41% ± 1.82%) followed by crude protein (10.52% ± 0.93%) and crude fat (1.68% ± 0.23%). The estimated macronutrient balance (i.e., realized nutrient niche) of the buffalo diet (20.5% protein: 72.8% carbohydrate: 6.7% lipid) was not significantly different than the average balance of all analyzed food items based on 95% confidence regions. Our study suggests that buffalo are likely macronutrient specialists, yet may be generalists in the sense that they feed on a wide range of food items to achieve a nutrient balance similar to that available in forage items. However, the four most frequently consumed items tended to be higher in protein energy than less frequently consumed foods, suggesting some preference for higher protein forage relative to relatively abundant carbohydrates. Although limited in scope, our study provides important information on the nutritional ecology of buffalo, which may be useful for the conservation and management of this endangered species.

## Graphical abstract

We explored for nutritional ecology of an endangered megaherbivore, the wild water buffalo (*Bubalus arnee*) in Nepal. Signs of wild water buffalo foraging were observed in the field, and samples of forage plants were collected for analysis of nutrient content. We report on the relative frequency of items foraged by wild water buffalo and their nutrient balance to investigate the multidimensional nutritional niche for this species.

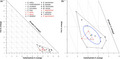

## 1 INTRODUCTION

Effective management of any population relies on adequate knowledge of the population's ecology, including population dynamics (e.g., density and growth), resource allocation, and habitat use. These aspects of a population's ecology are greatly influenced by the availability, abundance, and nutritional composition of food resources (Nielsen, Larsen, Stenhouse, & Coogan, 2017; Rode, Chapman, McDowell, & Stickler, 2006). Recent research has shed light on the importance of nutrient intake, including the balanced intake of multiple nutrients (i.e., carbohydrates, lipids, and proteins) in characterizing nutritional strategies of animals (Coogan et al., 2017; Takahashi, Rothman, Raubenheimer, & Cords, 2019). Nutrient balancing is the phenomenon by which animals homeostatically regulate their intake of foods to maintain a relatively consistent nutrient intake in the face of sometimes considerable variation in the nutritional composition of food items consumed (Simpson & Raubenheimer, 2012). Thus, intraspecific variation in diet composition due to environmental differences may not equate to significant differences in overall dietary nutrient composition. This phenomenon has been observed in geographically distinct populations of mountain gorillas, which regulated the composition of nutrients in their diets despite consuming different forage items (*Gorilla beringei*; Rothman, Plumptre, Dierenfeld, & Pell, 2007). Other species can tolerate widely different dietary nutrient compositions across their range, including omnivorous wild boars (*Sus scrofa*) and brown bears (*Ursus arctos*; Senior, Grueber, Machovsky‐Capuska, Simpson, & Raubenheimer, 2016; Coogan, Raubenheimer, Stenhouse, Coops, & Nielsen, 2018). Therefore, considerable insight into an animal's feeding strategies can be gained by examining diet at the level of both foods and nutrients in relation to food availability.

Researchers have moved beyond the traditional categories of dietary specialization (i.e., generalist versus specialist) in terms of the range of foods consumed to also encompass the nutritional and other characteristics (e.g., structural components) of foods in a multidimensional nutritional niche (Coogan, Raubenheimer, Zantis, & Machovsky‐Capuska, 2018; Machovsky‐Capuska, Senior, Simpson, & Raubenheimer, 2016). At one level, an organism's degree of specialization can be described in terms of the nutritional composition of foods consumed, where an animal with a diet consisting of foods varying broadly in nutritional composition can be considered a food composition generalist. At another level, the nutritional composition of a population's overall diet can be used to assess the realized nutritional niche of that population. The range of realized nutritional niches of a species can be used to infer that species fundamental nutritional niche. Species with a wide range of realized niches (i.e., large fundamental nutritional niche) may be considered nutrient generalists. Conversely, species with a narrow range of realized niches (i.e., small fundamental nutrient niche) can be considered nutrient specialists. Finally, the multidimensional nutritional niche framework also considers the physical and non‐nutritional properties of foods, such that an animal with the ability to consume a variety of foods that vary structurally can be considered a food exploitation generalist.

The wild water buffalo (*Bubalus arnee*; hereafter referred to as “buffalo”) is a species that has been the subject of intensive management over the previous 60 years, and is listed by the IUCN as *Endangered* (Kaul, Williams, Rithe, Steinmetz, & Mishra, 2019). Throughout their current range, buffalo select low‐lying alluvial grassland habitats (Heinen & Paudel, 2015). While little research has explicitly studied the diet of buffalo, feeding observations suggest they are predominantly grazers, but have been observed foraging on forbs and browse, especially new growth (Choudhury, 2014). In Thailand, 45% of buffalo diet was composed of 3 grass species (Chaiyarat, 2002). A population of buffalo in Assam, India, has regularly been observed foraging on water hyacinth (*Eichhornia crasspies*), an invasive forb that has become common in the freshwater systems of the area (Choudhury, 2014). Buffalo are also known to raid anthropogenic crops such as rice, sugarcane, and jute from agricultural lands on the fringes of their home range, which has led to buffalo–human conflicts in and around protected areas (Choudhury, 2014).

In Nepal, buffalo were restricted to the Koshi Tappu Wildlife Reserve (KTWR) until 2017 when a second population was established in Chitwan National Park. To date, research into the ecology of the KTWR buffalo has focused on population growth and genetic integrity. Censuses of the KTWR population have been conducted sporadically since 1976. These censuses have described an average annual population growth rate of 3.3% throughout that time (Dahmer, 1978; Heinen, 1993; Heinen & Kandel, 2006; Heinen & Singh, 2001; Khatri, Shah, & Mishra, 2012). This growth rate is consistent with population growth rates of other large, long‐lived ungulates that have adequate habitat and are not subject to predation pressure (Clutton‐Brock, 1989; Heinen & Paudel, 2015). The most recent census conducted in 2018 reported 441 individuals in the KTWR's buffalo population (KTWR, 2018). Females are typically found in herds of 13–17, and bachelor herds have been observed being comprised of 9–12 individuals (Heinen, 1993).

In this paper, we sought to further the understanding of the nutritional ecology of buffalo in the KTWR to facilitate its conservation and management. We used multidimensional nutritional niche concepts to evaluate the foraging choices of free‐ranging buffalo during the autumn in the KTWR. First, we identified plant species that were foraged by buffalo. Then, to understand aspects of the food exploitation level of their nutritional niche, we evaluated the relative frequency (RF) of graminoids, forbs, and browse foraged by buffalo. Next, we explored the nutrient balance of foraged species to gain insight into the degree of nutrient specialization and the realized nutrient niche for buffalo in the KTWR. We predicted that the realized nutrient niche for buffalo would be highest in the proportion of carbohydrate energy, moderate in protein, and with the lowest proportion for lipid, in keeping with the dietary nutrient balance of other Nepalese herbivores (Aryal, Brunton, et al., 2015; Aryal, Coogan, Ji, Rothman, & Raubenheimer, 2015; Koirala et al., 2019).

## 2 MATERIALS AND METHODS

### 2.1 Study area

The KTWR was established in 1976 to preserve the last Nepalese population of buffalo and act as a migratory bird sanctuary (Heinen & Paudel, 2015)*.* The KTWR lies on the floodplains of the Saptakoshi River in the South‐East Terai region of Nepal (Sah, 1997). The reserve has subtropical climate, with an elevation ranging between 75 and 100 m above sea level. Nepal has four climatic seasons, including spring (March–May), summer (June–August), autumn (September–November), and winter (December–February). The reserve covers a 175‐km^2^ core area with a 173 km^2^ buffer zone. The KTWR incorporates two municipalities of the Saptari district, two municipality of the Udayapur district, and one municipality and one rural municipality of Sunsari district, with a combined population of 84,423 people among 14,865 households (KTWR, 2018). The KTWR is mostly comprised of alluvial grasslands (56%) and large sand/gravel deposits (22%) with some forest (1%), lakes and ponds (0.01%), marshes and swamps (6%), rivers and streams (10%), and, in the buffer zone, agricultural land (5%) (Chettri, Uddin, Chaudhary, & Sharma, 2013). In 2009, a botanical survey described 670 species of vascular plants in the reserve (Siwakoti, 2009). Natural predators of buffalo (e.g., tigers, *Panthera tigris*; leopards, *Panthera pardus*; and dholes, *Cuon alpinus*) have been extirpated from the KTWR for at least 40 years (Heinen & Paudel, 2015). Likewise, large mammalian herbivores such as gaur (*Bos gaurus*) and blue bull (*Boselaphus tragocamelus*) have declined in numbers and are now rare in the KTWR.

### 2.2 Field methods

We conducted field surveys during the autumn (November 2017) following the hot, wet summer monsoon season and avoiding the cool, dry winter season. During the winter vegetation, dies during the dry period and summer monsoon floods limit buffalo to foraging on islands and in croplands (Chettri et al., 2013). Therefore, we conducted surveys for buffalo foraging during November, when the KTWR buffalo have the greatest access to forage and travel widely throughout their study area.

**FIGURE 1:**
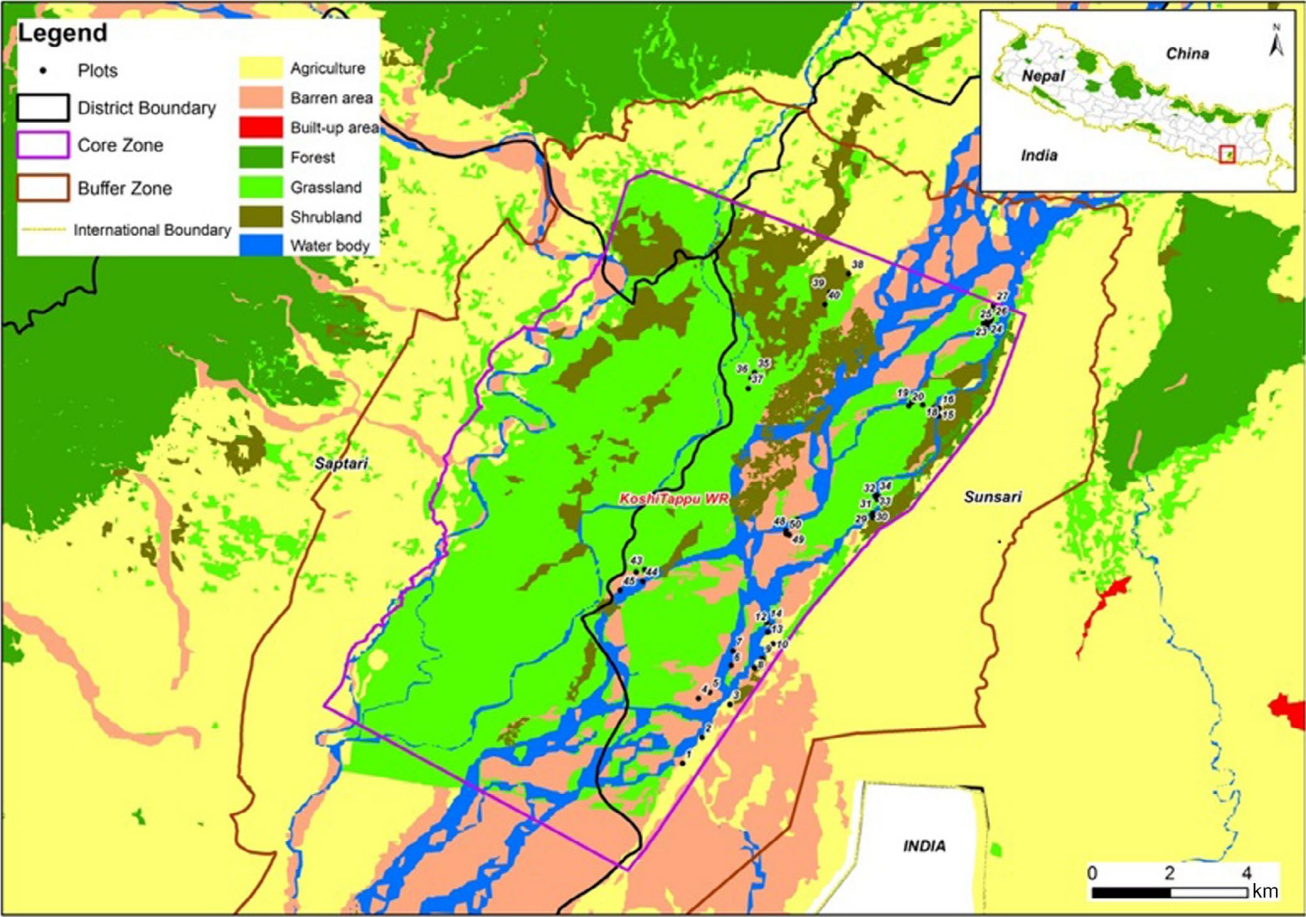
Koshi Tappu Wildlife Reserve, Nepal, and the distribution of foraging plots established in November 2017. Foraging plots are numbered from 1 to 50

We identified and sampled vegetation in bison foraging plots following a modified version of Ngoti (2017). We established 50 foraging plots (5 m × 5 m) throughout the KTWR in areas where buffalo are typically observed, and where fresh buffalo dung and signs of foraging were present (Figure 1). We located and identified buffalo dung with the help of a local KTWR guides who had knowledge of buffalo ecology and behavior. We were careful to visually identify dung based on its physical characteristics to prevent misidentification of domestic cattle dung as belonging to buffalo. In addition, rarity of other large herbivores in the park facilitated buffalo dung identification. We established the square 5 m × 5 m foraging plots using a measuring tape, where the perimeters were set using wooden pegs in each of the four corners with plastic ropes delineating each of the four sides of the plot. Once the plot was set, we recorded plot‐level field data, such as plot ID number, date, latitude, longitude, habitat type, presence or absence of cattle, and existing plant species. Plants that were grazed, browsed, or debarked were carefully identified and recorded during data collection following a modified protocol from Ngoti (2017). We did not attempt to quantify the amount or proportion of foraging on different species in plots, rather we simply identified whether a species was foraged or not and that species functional foraging group (i.e., forbs, graminoids, or browse). Plants were identified with the help of our local guide as well as local residents familiar with the flora in the park.

We collected representative samples of foraged plants of the same species from nearby plants within the foraging plots that were not foraged. We therefore assumed that the plants we collected for analysis had the same nutritional properties as foraged plants. We clipped plant samples for collection in a manner that mimics the way the plant was foraged by buffalo at that site. In general, we clipped grasses above the organic debris covering the ground, and leaves of browse items were clipped up to approximately 1.8 m off the ground. We sealed samples of each plant species in airtight plastic bags and transported for nutrient content analysis. In field, plant sample identification was verified at the Department of Plant Resources herbarium in Kathmandu, Nepal.

### 2.3 Diet and nutritional analysis

After collecting plants, and before conducting nutritional analysis, we estimated the buffalo's diet by calculating the frequency and relative frequency (RF) of all foraged species using the following equations from Fracker and Brischle (1944):

Frequency of speciesx(\% )=(Number of plots where speciesxwas foraged/Total number of foraging plots)×100

Relative frequency (\% )=(Frequency of speciesx/Total frequency of each foraged species)×100

Due to research constraints, we selected 16 plant species for nutritional analysis that were foraged in at least 5 plots (i.e., RF > 2%). Samples of the same species from different plots were mixed together to yield a composite sample, from which we used 200–400 g for nutritional analysis. Thus, we were unable to consider intraspecific variation in nutritional composition between plants and nutritional estimates can be considered representative of the average nutritional composition of the forage species of buffalo.

We used proximate nutritional analysis to estimate the percent moisture, ash, crude fat, crude protein, crude fiber, and available carbohydrate content of each foraged plant species. Samples of each species were sent to the Nepal Environmental and Scientific Services laboratory in Kathmandu, Nepal, for analysis. Association of Official Agricultural Chemists (AOAC) methods were used to determine moisture, ash, crude fat, and crude fiber (AOAC, 2006). Nitrogen content was determined by the micro Kjeldahl method and multiplied by a factor of 6.25 to estimate crude protein content (Pearson, 1976). Available carbohydrates were determined by the subtraction method (i.e., available carbohydrate (%) = 100% ‐ ash (%) ‐ crude fat (%) ‐ crude fiber (%) ‐ crude protein (%) – moisture (%) (Merrill & Watt, 1973).

We evaluated differences in nutrient content of plant species using Student's *t* tests (*α* = 0.05; Ramsey & Schafer, 2002). To assess differences in nutrient content between species, we calculated mean nutrient content (%) and conducted a one‐way ANOVA and a *post hoc* Tukey's HSD test using the Statistical Package for the Social Sciences (SPSS version 2.0; IBM Corp. 2011).

To evaluate plant nutrient balance and the realized nutrient niche of buffalo diet during November, we generated right‐angled mixture triangles (RMT) to plot the nutrient composition of the most frequently foraged plant species (Machovsky‐Capuska et al., 2016; Raubenheimer, 2011). To evaluate nutrient balance, we first converted macronutrients to units of metabolizable energy using conversion factors of 9 kcal/g for lipids and 4 kcal/g for carbohydrates and proteins (Coogan, Raubenheimer, Stenhouse, & Nielsen, 2014; Merrill & Watt, 1973). Following conversion, the metabolizable energy values for each macronutrient in individual food items were summed together and then expressed as a percentage of the sum of total metabolizable energy. Food items were plotted as Cartesian points within RMTs based on their metabolizable energy content. We drew convex hull polygons (Wijeweera & Kodituwakku, 2018) around the foraged plants plotted in the RMT to visually evaluate the range (or breadth) of macronutrient compositions in food items of buffalo, and thus the degree of macronutrient generalism or specialization exhibited by buffalo based on their foraging preferences. We then weighted the macronutrient proportions of forage items by their RF to estimate the buffalo's November diet, their realized macronutrient niche for that period (Machovsky‐Capuska et al., 2016).

## 3 RESULTS

We identified 54 plant species (2 species were unidentified) that were foraged within 50 buffalo foraging plots (Table 1). Grazing, browsing, debarking, and stem chewing were the primary modes of foraging observed in the plots. Of the 54 plant species, 16 had an RF > 2% and occurred in at least five foraging plots (Table 1). Furthermore, these 16 plant species accounted for roughly two‐thirds (67.73%) of the total foraging of recorded plants. The four species with the highest RF included *Typha elephantina, Saccharum spontaneum, Tamarix dioica,* and *Phragmites karka*, which together accounted for >25% of all foraged species. Of the 38 remaining species, 18 were recorded as foraged in only one plot.

**TABLE 1:** Plant species foraged by wild water buffalo (*n* = 54) and the relative frequency (RF %) that foraging was observed for each species

Plants	FG	RF %	Plants	FG	RF %
*Typha elephantina*	F	9.09	*Justicia adhatoda*	B	0.91
*Saccharum spontaneum*	G	6.82	*Mimosa pudica*	B	0.91
*Tamarix dioica*	B	5.45	*Oryza rufipogon*	G	0.91
*Phragmites karka*	G	5.00	*Alternanthera paronychioides*	F	0.91
*Cynodon dactylon*	G	4.55	*Vetiveria zizanioides*	G	0.91
*Eleusine indica*	F	4.55	*Amaranthus tricolor*	F	0.91
*Mikania micrantha*	F	4.55	*Elephantopus scaber*	F	0.91
*Cyperus rotundus*	G	3.64	*Breea arvensis*	F	0.91
*Bulbostylis barbata*	G	3.64	*Bidens biternata*	F	0.91
*Diplazium esculentum*	F	3.64	*Cajanus cajan*	B	0.45
*Marsilea minuta*	F	3.18	*Oxystelma esculentum*	F	0.45
*Sida cordifolia*	B	2.73	*Eulaliopis binata*	G	0.45
*Chrysopogon aciculatus*	G	2.73	*Buddleja asiatica*	B	0.45
*Euphorbia hirta*	F	2.73	*Alternanthera sessilis*	F	0.45
*Cissus quadrangularis*	F	2.73	*Ageratum conyzoides*	F	0.45
*Imperata cylindrica*	G	2.73	*Tinospora sinensis*	F	0.45
*Zizyphus mauritiana*	B	1.82	*Crotalaria alata*	F	0.45
*Centella asiatica*	F	1.82	*Pseudognaphalium luteoalbum*	F	0.45
*Lathyrus aphaca*	F	1.82	*Asclepias curassavica*	B	0.45
*Bidens pilosa*	F	1.82	*Desmostachya bipinnata*	G	0.45
*Achyranthes aspera*	F	1.36	*Biophytum sensitivum*	B	0.45
*Sphaeranthus indicus*	F	1.36	*Abrus precatorius*	F	0.45
*Adiantum phillipense*	F	1.36	*Leersia hexandra*	G	0.45
*Rauwolfia serpentina*	B	1.36	*Ceratopteris thalictroides*	F	0.45
*Caesalpinia tora*	B	1.36	*Saccharum officinarum*	G	0.45
*Thysanolaena maxima*	G	0.91	Unidentified 1	G	0.45
*Calotropis gigantea*	B	0.91	Unidentified 2	G	0.45

Note

The 16 most frequently foraged plants are shaded in grey and were used in analysis of nutrient composition of wild water buffalo diets. The functional forage group (FG) for each species is also listed as graminoid (G), forb (F), or browse (B).

**FIGURE 2:**
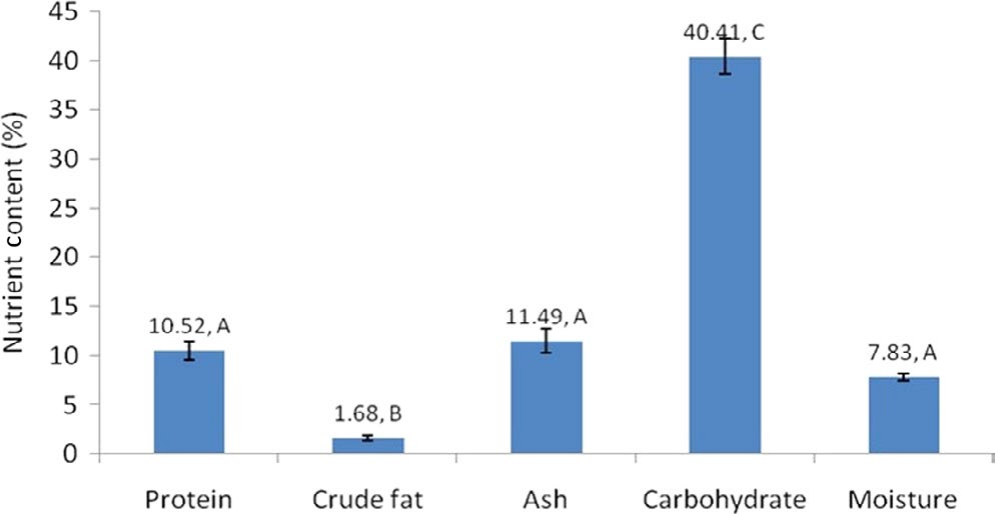
Mean content of plant components in species foraged by wild water buffalo. The mean content of protein, ash, and moisture is not significantly different as shown by “A,” the mean content of the crude fat is significantly lower than other nutrients shown by “B,” and the mean content of carbohydrate is significantly higher shown by “C.”

**FIGURE 3:**
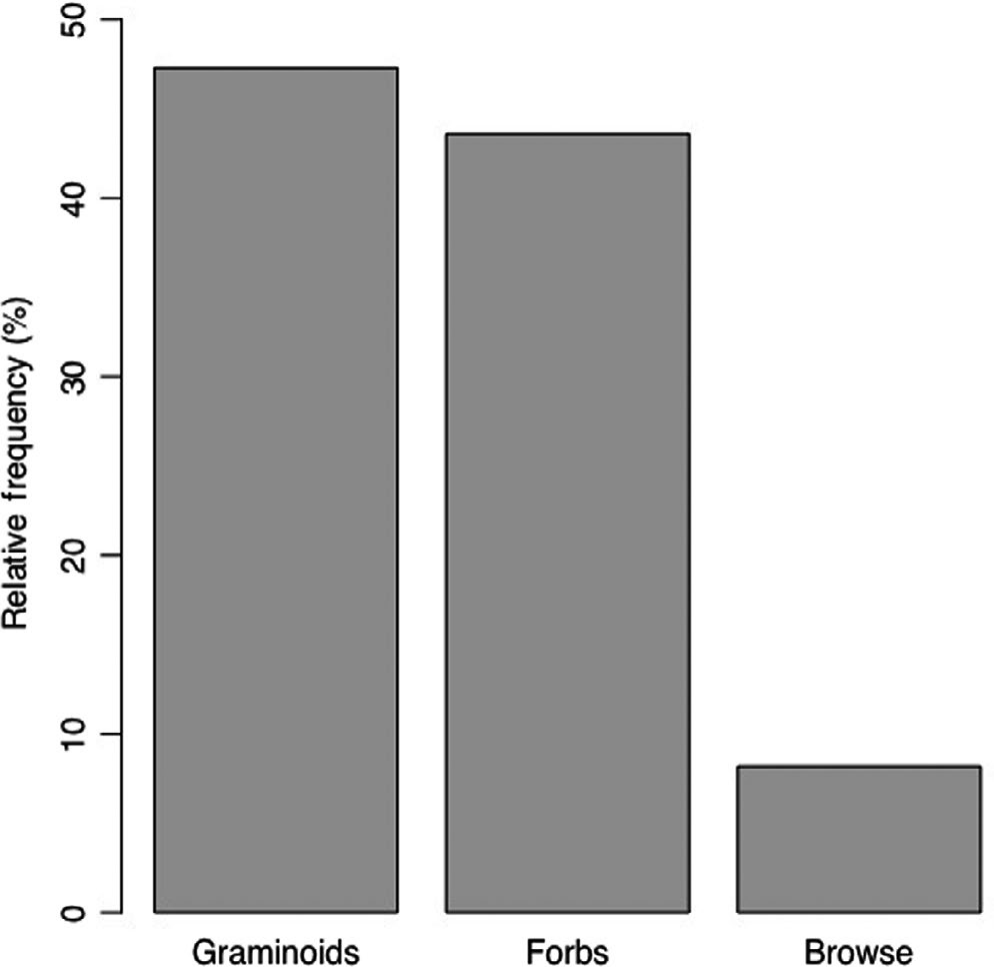
The mean relative frequency at which graminoids, forbs, and browse species were foraged by wild water buffalo (*Bulbalus arnee*) in the Koshi Tappu Wildlife Reserve

Carbohydrates had the highest mean value among nutrients in plants foraged by buffalo (40.41% ± 1.82%) followed by ash (11.49% ± 1.23%), crude protein (10.52% ± 0.93%), moisture (7.83% ± 0.32%), and crude fat (1.68% ± 0.23%) (Table 2, Figure 2). The range of carbohydrate composition among plants was also higher (29.24%–55.61%) than other nutrients. *Marsilea minuta* had the highest carbohydrate content (55.61%), followed by *Chryspogon aciculatus* (52.36%), *Cynodon dactylon* (46.73%), and *Imperata cylindrica* (45.34%). *Tamarix indica* had the highest proportion of protein (15.06%), followed by *Euphorbia hirta* (15.00%), *Eleusine indica* (14.06%), and *S. spontaneum* (14.00%). *Euphorbia hirta* had the greatest amount of crude fat (4.10%), then *Mikania micarantha* (2.81%), *Sida cordifolia* (2.76%), and *Cissus quadrangularis* (2.31%). The one‐way ANOVA showed the mean nutrient content in these plant samples was significantly different (*F* = 193.91, *p* = .000, *α* = 0.05; Figure 2). Additionally, we found that forbs and graminoids were foraged 2.8 and 2.0 times more frequently than browse items (Figure 3).

**TABLE 2:** Results of the proximate analysis describing the nutritional composition as a percentage of the 16 most frequently foraged plants of wild water buffalo

Plants	Crude protein	Crude fat	Ash	Available carbohydrate	Moisture	Crude fiber
*Bulbostylis barbata*	13.31	1.42	9.60	43.91	8.50	23.26
*Chryspogon aciculatus*	4.63	1.26	13.30	52.36	7.30	21.15
*Cissus quadrangularis*	10.25	2.31	21.20	34.91	8.10	23.23
*Cynodon dactylon*	8.18	0.45	11.70	46.73	6.80	26.14
*Cyprus rotundus*	6.00	1.32	6.10	41.91	7.70	36.97
*Diplazium esculentum*	7.56	1.30	11.50	34.78	10.80	34.06
*Eleusine indica*	14.06	0.48	21.50	33.70	7.40	22.86
*Euphorbia hirta*	15.00	4.10	13.30	34.25	8.40	24.95
*Imperata cylindrica*	3.88	1.42	5.90	45.34	6.40	37.06
*Marsilea minuta*	9.06	1.32	9.20	55.61	9.20	15.61
*Mikania micarantha*	11.69	2.81	9.60	32.04	8.60	35.26
*Phragmites karka*	10.38	1.18	12.80	39.21	5.60	30.83
*Saccharum spontaneum*	14.00	1.11	3.50	29.24	8.10	44.05
*Sida cordifolia*	13.38	2.76	14.30	41.15	8.50	19.91
*Tamarix dioica*	15.06	1.55	12.70	42.24	7.60	20.85
*Typha elephantina*	11.88	2.05	7.60	39.16	6.20	33.11

**FIGURE 4:**
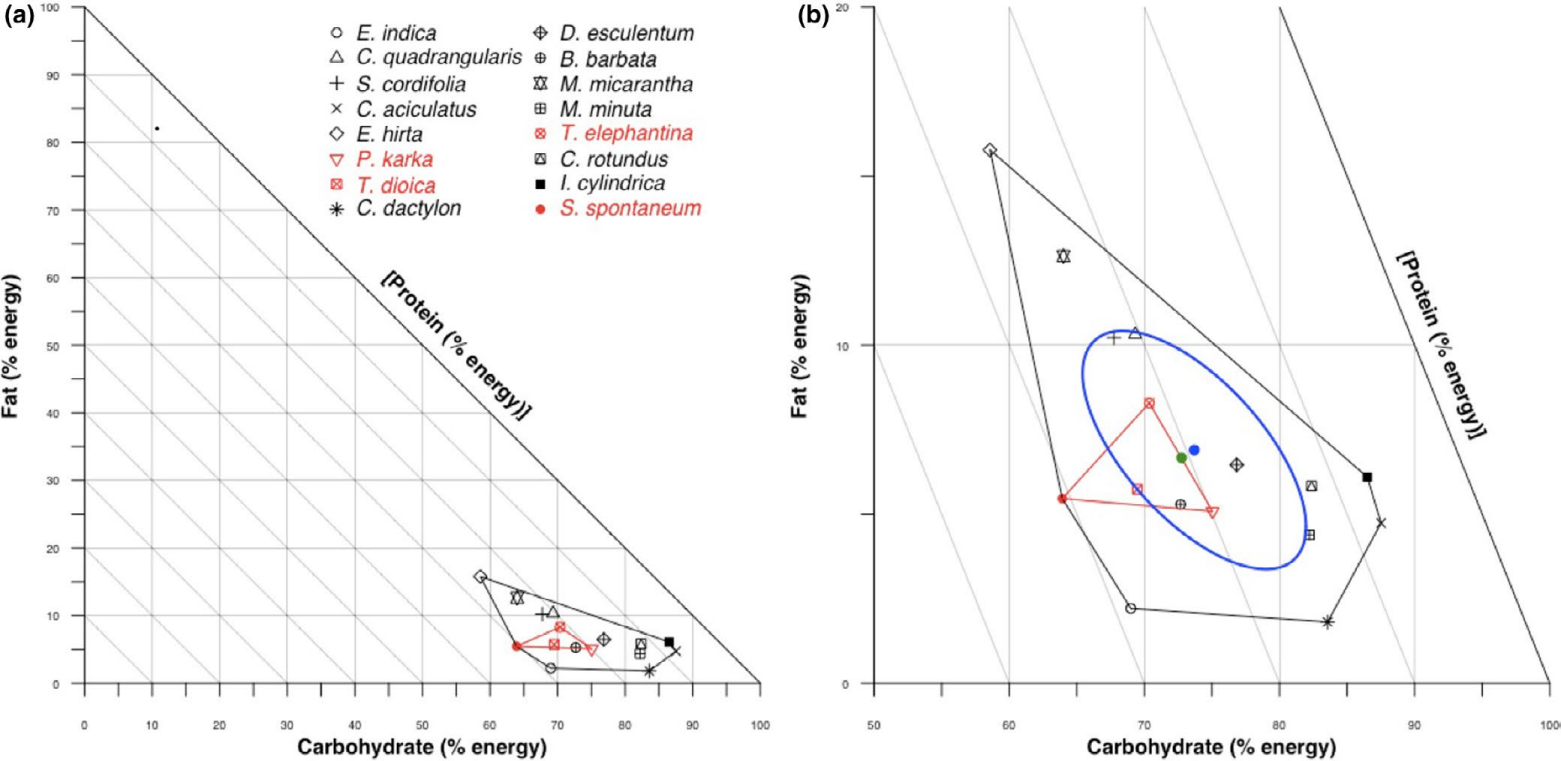
(a) Right‐angled mixture triangle showing the relative proportions of metabolizable energy from carbohydrates, lipids, and proteins in the 16 most common plant species foraged by wild water buffalo (*Bulbalus arnee*) in the Koshi Tappu Wildlife Reserve, Nepal. The black convex hull polygon represents the mixture space of food items consumed by wild water buffalo. The red polygon highlights the nutrient mixture space of the four species that made up greater than 25% of foraged plants. (b) A closer look at the region of nutrient space occupied by the most commonly foraged plant species of wild water buffalo. The blue dot (with accompanying 95% confidence region outlined in blue) is the mean nutrient proportion of all forage items and represents what the nutrient composition of the diet would be if wild water buffalo foraged randomly on these species. The green dot is an estimate that describes the realized nutrient proportions of the wild water buffalo's diet as estimated by weighting the nutrient proportions of food items by the relative frequency (RF) they were foraged upon. Because both the mean nutrient proportion of food items and the weighted diet nutrient proportions lie within the 95% confidence region, we can infer that there is no significant difference between them

Our RMTs revealed that the 16 most frequently foraged species by buffalo ranged in percent metabolizable energy from 58% to 88% for carbohydrate, 7% to 31% for protein, and 1% to 16% for lipids (Figure 4). The four species that accounted for greater than 25% of the foraged plants occupied a nutrient space that was relatively higher in protein concentration and lower in carbohydrates than the 12 other species. However, the mean nutrient composition of the 16 most frequently consumed forage plants (19.4% protein: 73.7% carbohydrate: 6.9% lipid) was not significantly different than the estimated nutrient balance (20.5% protein: 72.8% carbohydrate: 6.7% lipid) of their weighted diet (i.e., the estimated dietary nutrient proportions of forage items weighted by the RF of food items consumed) based on the 95% confidence region (Figure 4).

## 4 DISCUSSION

Our estimate of KTWR buffalo's realized macronutrient niche during November supports our prediction that buffalo occupy a macronutrient niche high in carbohydrates, moderate in protein, and low in lipids. While there was variation between the nutrient compositions of foraged plants, the proportions of nutrient energy in plant species were confined to a relatively restricted region of nutrient space typical of herbivore diets. For example, our results for buffalo are similar to the nutrient balance described for other Nepalese herbivores, such as blue sheep (*Psuedois nayaur*; Aryal, Coogan, et al., 2015), Asian elephant (*Elephas maximus*; Koirala et al., 2019), and Himalayan marmot (*Marmota himalayana;* Aryal, Brunton, et al., 2015). Given the relatively small nutrient niche breadth of buffalo we observed, it is likely that they are food composition specialists, especially when compared to omnivorous species such as brown bear and wild boar, which can consume a range of high‐carbohydrate, high‐protein, and high‐lipid foods (i.e., food composition generalists; Coogan, Raubenheimer, Stenhouse, et al., 2018; Senior et al., 2016). However, buffalo may have some tendency to be nutrient generalists capable of tolerating a range of diet compositions within a relatively narrow herbivorous fundamental nutrient niche, contingent on the availability of food items. Therefore, the buffalo may have a less‐selective generalist herbivore foraging strategy whereby their dietary preferences and regulatory systems have adapted to nutrient proportions generally available in plant foods within their range.

There are a few caveats to our analysis. Our nutritional analysis is conducted on a limited number of food items and relatively short period of sample collection, and thus, longer‐term studies may yield more dietary variety and differences in diet estimates between seasons. Likewise, dietary differences between sexes and life‐history stages in buffalo may exist. We also acknowledge that because our selection of survey plots was based on signs of buffalo presence and foraging (not observation of buffalo foraging) that other herbivores may have foraged plants within survey plots. However, populations of other large ungulate herbivores in the KTWR are sparse (Khatri, Shah, Shah, & Mishra, 2010) and the guides employed to identify signs of buffalo have expertise in distinguishing buffalo sign from other local herbivores. Furthermore, we did not look at metabolizable energy from digestible fiber (e.g., Aryal, Brunton, et al., 2015), which has been shown to influence food selection in other herbivores (Aryal, Coogan, et al., 2015). Yet, given the consistency with other nutrient balance studies of herbivores, we consider that the nutrient niche breadth and realized nutrient niche fall within expected values.

Buffalo do show some degree of flexibility in their food exploitation niche by foraging upon plants that vary in their physical characteristics. For example, we did not find a significant difference between the RFs of individual species of graminoids, forbs, and browse items. However, when pooled by forage group, graminoids and forbs were foraged between two to three times as frequently as browse items, suggesting that buffalo are intermediate feeders like other megaherbivores such as American Bison (*Bison bison*; Leonard, Perkins, Lammers, & Jenks, 2017). One of the four most frequently foraged plants, French tamarix (*T. dioica*), was a shrub, and there have been a number of reports of buffalo foraging on the leaves of saplings such as *Shorea robusta* and *Brideliea retusa* (Choudhury, 2014). These findings suggest that buffalo do have some plasticity in their diet. Further research should investigate when buffalo consume browse items, as they may select them during particular phenophases (i.e., an observable stage in the annual life cycle of a plant) or to compensate for a deficiency in a particular nutrient.

Our observations of buffalo foraging indicated that *T. elephantina* (forb), *S. spontaneum* (graminoid), *T. dioica* (browse), and *P. karka* (graminoid) were dominant foods in the diet of buffalo. This result conflicts with the results of Chaiyarat (2002), the only other study that explicitly tried to describe the diet of buffalo, but in Thailand. Chaiyarat (2002) found that 90% of buffalo diet was composed of grasses based on fecal analysis in Huai Kha Khaeng Wildlife Sanctuary, Thailand. However, that diet was analyzed using microhistology, which has been critiqued as showing a positive bias toward grasses that tend to pass through the digestive system intact and are therefore more readily identified in fecal analyses than forb or browse species (Varva & Holecheck, 1980). Additionally, the author did acknowledge that forbs and shrubs were present in buffalo diets (Chaiyarat, 2002). Our study is the first to describe forbs as a dominant part of buffalo diets. Modern methods of diet analysis (e.g., DNA barcoding) are illuminating the importance of forbs in other mega‐herbivore diets such as North American bison (*Bison bison*; Craine, Towne, Miller, & Fierer, 2015; Leonard et al., 2017) and may be useful to employ in future studies of buffalo.

Despite the limitations of this study, our research contributes important knowledge on the feeding preferences and nutritional content of plants foraged upon by endangered buffalo in Nepal, a species for which relatively limited information is available. The results presented here may be used to inform conservation and management strategies for this species in wild. For example, understanding why buffalo forage on particular species can enlighten managers to foraging and habitat preferences of buffalo in the KTWR, and elsewhere. Additionally, our research may aid in the understanding of why buffalo target specific anthropogenic crops, which likely have similar nutritional properties to the preferred forage species. Habitat management for buffalo conservation in Nepal should consider the availability of key forage species (e.g., *T. elephantina*, *S. spontaneum*, *T. dioica*, and *P. karka*), sufficient quantities of which may help reduce crop depredation. Furthermore, the aforementioned species are critical to other Nepalese mega‐herbivores, such as the greater one‐horned rhinoceros (*Rhinoceros unicornis*), which had over 40% of its diet composed of *S. spontaneum* and *P. karka* in Royal Bardia National Park (Steinheim, Wegge, Fjellstad, Jnawali, & Weladji, 2005). These species dominate Nepalese tallgrass floodplains and the findings that two endangered herbivores utilize them as a critical component of their diet emphasizes the importance of protecting tallgrass floodplains in Nepal.

Future research should expand upon this study by lengthening the period of observation and examining diet using different techniques. Likewise, elucidating the nutritional intake target (sensu Simpson & Raubenheimer, 2012) and other physiological and behavioral aspects of the species nutritional ecology would further aid in buffalo management and conservation. Additionally, considering how other biotic factors (e.g., predation) influence the amount of time and effort put into foraging by buffalo may yield additional insight. For example, a particularly interesting line of research would be to compare the foraging behavior of buffalo in KTWR, where predators have been extirpated, to buffalo in Chitwan National Park (CNP), which still houses tigers. Theory predicts that buffalo will spend less time at a particular foraging station, more time being vigilant, and an overall lower quality diet while foraging on a “landscape of fear” in CNP (Hernandez & Laundre, 2005).

## CONFLICT OF INTEREST

None declared.

## AUTHOR CONTRIBUTIONS

**Tej Kumar Shrestha:** Conceptualization (equal); data curation (equal); formal analysis (equal); investigation (lead); methodology (equal); project admin (lead); resources (lead); validation (equal); writing–original draft (lead); writing–review and editing (equal). **Lee J. Hecker:** Conceptualization (equal); data curation (equal); formal analysis (equal); methodology (equal); validation(equal); writing–review and editing (lead). **Achyut Aryal:** Conceptualization (equal); methodology (equal); supervision (equal); validation (equal); writing–original draft (supporting); writing–review and editing (equal). **Sean C. P. Coogan:** Conceptualization (equal); data curation (equal); formal analysis (equal); methodology (equal); supervision (equal); validation (equal); writing–review and editing (equal).

## Data Availability

A complete list of foraged species with relative frequency statistics and macronutrient content of the 16 most frequently foraged species can be retrieved via Dryad: https://doi.org/10.5061/dryad.vt4b8gtp0.
